# How can we obtain truly translational mouse models to improve clinical outcomes in schizophrenia?

**DOI:** 10.1242/dmm.049970

**Published:** 2022-11-28

**Authors:** Steven J. Clapcote

**Affiliations:** School of Biomedical Sciences, University of Leeds, Leeds LS2 9JT, UK

**Keywords:** Biological psychiatry, Mouse models, Schizophrenia

## Abstract

Schizophrenia is a serious mental illness affecting 0.7% of the world’s population. Despite over 50 years of schizophrenia drug identification and development, there have been no fundamental advances in the treatment of schizophrenia since the 1980s. Complex genetic aetiology and elusive pathomechanisms have made it difficult for researchers to develop models that sufficiently reflect pathophysiology to support effective drug discovery. However, recent large-scale, well-powered genomic studies have identified risk genes that represent tractable entry points to decipher disease mechanisms in heterogeneous patient populations and develop targeted treatments. Replicating schizophrenia-associated gene variants in mouse models is an important strategy to start understanding their pathogenicity and role in disease biology. Furthermore, longitudinal studies in a wide range of genetic mouse models from early postnatal life are required to assess the progression of this disease through developmental stages to improve early diagnostic strategies and enable preventative measures. By expanding and refining our approach to schizophrenia research, we can improve prevention strategies and treatment of this debilitating disease.

## Teaser

**Summary:** This Editorial explores how recent advances in schizophrenia genomics and longitudinal assessment of prodromal signs can be harnessed to develop and study mouse models that sufficiently reflect schizophrenia pathophysiology and support effective drug discovery.

## The need for better treatments for schizophrenia

Schizophrenia is a chronic mental illness that afflicts ∼0.7% of the world’s population (MacDonald and Schulz, 2009). Clinical manifestations of schizophrenia include positive (psychotic) symptoms, such as hallucinations, delusions, bizarre thoughts and paranoia; negative symptoms, such as social withdrawal, avolition, alogia and apathy; and cognitive deficits, including impairments in executive functions, working memory and attention (Picchioni and Murray, 2007). Symptoms emerge after puberty – typically in late adolescence or early adulthood (Häfner et al., 1994) – and cause significant impairment in social and occupational functioning, with substantial individual, family and societal costs (Carr et al., 2003).

Current treatments with typical (first-generation) and atypical (second-generation) antipsychotics, which share a common mechanism of action in antagonizing the dopamine D_2_ receptor, mainly attenuate the positive symptoms. However, they do not produce meaningful improvements in negative and cognitive symptoms, both of which greatly affect social and occupational functioning in patients (Girgis et al., 2019). Reducing the duration of untreated psychosis through early detection and pharmacological intervention is associated with enhanced treatment response, functional improvement and maintenance of symptom remission (Perkins et al., 2005). Even so, ∼30% of patients – with so-called treatment-resistant schizophrenia – experience no therapeutic benefit from first-line antipsychotics. This leaves clozapine as the sole medicinal option, which is associated with life-threatening side effects that require strict monitoring (Correll and Howes, 2021).

The suboptimal current treatment options, together with a median annual recovery rate of only 1.4% (Jääskeläinen et al., 2013), pose a challenge for the research community. However, despite more than 50 years of schizophrenia drug discovery, there have been no fundamental advances in the treatment of schizophrenia since the 1980s (Conn and Roth, 2008; Girgis et al., 2019). This highlights the urgent need for a clearer understanding of the pathogenesis of schizophrenia, so that better treatments can be developed based on testable hypothesis-driven research, in which preclinical models are important tools (Campbell and Granato, 2020; Canetta and Kellendonk, 2018).

## Mouse models based on early candidate genes: example of *DISC1*

Psychiatry has lagged behind other medical disciplines in mechanistic understanding, and the development of valid biomarkers and improved treatments. A main critique of drug discovery approaches is that new treatments cannot be developed while the underlying causes of schizophrenia remain incompletely understood (Conn and Roth, 2008). As schizophrenia is predominantly a genetic disorder – with heritability estimated to be ∼80% (Sullivan et al., 2003) – genomic studies of schizophrenia patients are a rational approach to obtain novel mechanistic insights and prospective drug targets (Insel and Collins, 2003). This is envisaged to pave the way for the development of mechanistically targeted drugs with improved therapeutic efficacy.

Introduction of human genetic variants associated with schizophrenia into model organisms is an important strategy to understand their functional relevance, explore the underlying pathophysiology, and evaluate candidate therapies. Selecting genes for experimental manipulation in preclinical models is dependent on the human genetic evidence available at the time. Prior to the current era of large-scale genome-wide association studies (GWAS) and exome studies, our knowledge of the genetic basis of schizophrenia was much more limited.

A report in the year 2000 identified a translocation that truncated the gene disrupted-in-schizophrenia 1 (*DISC1*) and co-segregated with several psychiatric illnesses in a large Scottish pedigree (Millar et al., 2000). Following on from this, candidate gene studies reported *DISC1* to be a putative susceptibility gene in patient populations with various psychiatric disorders (Harrison and Weinberger, 2005). Although the evidence for association of *DISC1* with schizophrenia had been inconclusive, many researchers, including me, began to explore the biology of *DISC1* in relation to psychiatric illness, often using mouse models (Clapcote et al., 2007; Jaaro-Peled, 2009; Tomoda et al., 2016). Currently, the Mouse Genome Informatics website lists 15 *Disc1* mutant and six *DISC1* transgenic mouse lines (Blake et al., 2021).

Multiple studies have been published in prominent scientific journals (Brandon and Sawa, 2011). Research linking *DISC1* with cortical development and cyclic AMP signalling (Kamiya et al., 2005; Millar et al., 2005) featured among the top five scientific breakthroughs in 2005, according to the journal *Science* (Anonymous, 2005). *DISC1* was referred to as ‘one of the most compelling risk genes for schizophrenia’ (Wang et al., 2008) and ‘a key susceptibility gene for schizophrenia’ (Ming and Song, 2009). However, it is now widely accepted that *DISC1* is unlikely to be an important genetic risk factor for schizophrenia because no genetic study beyond the Scottish pedigree has met contemporary significance thresholds for rare exonic variation, rare copy number variation or common variation (Farrell et al., 2015; Niwa et al., 2016; Sullivan, 2013).

A considerable amount of biological and mouse model data on other putative susceptibility genes identified in the pre-GWAS era, such as *COMT*, *DTNBP1* and *NRG1*, have also been reported (Harrison and Weinberger, 2005; Wang et al., 2021). However, none of these genes is now supported by contemporary empirical evidence with rigorous standards for significance (Farrell et al., 2015). Our inadequate understanding of the aetiology of schizophrenia thus, at least partly, explains why no new therapeutic options have been developed despite decades of research with preclinical models (Canetta and Kellendonk, 2018; Conn and Roth, 2008).

## Large genomic studies identify genes for further investigation

We are now in the era of large-scale genomic studies for schizophrenia, which is providing unprecedented opportunities to gain new insights into the biological basis of schizophrenia. GWAS investigate more than a million common genetic variants across the human genome to determine their association with a disease. It has been argued that common small-effect risk variants identified in GWAS – which explain around one-third of the genetic liability to schizophrenia (Legge et al., 2021) – may provide solid therapeutic targets to inform precision medicine approaches (Gandal et al., 2016). A landmark study reported common variants that increase *C4A* expression in the brain in correlation with increased schizophrenia risk (Sekar et al., 2016), possibly through enhanced synaptic pruning (Yilmaz et al., 2021). Despite this exciting discovery, deciphering the genes and associated mechanisms influenced by common variants in schizophrenia has been difficult.

Compared with the hundreds of common small-effect, mainly non-coding, variant loci identified in GWAS, rare large-effect coding variants affecting specific genes lend themselves to experimental investigation in model organisms by being more interpretable and tractable. However, by definition, rare variants – i.e. those with a minor allele frequency of <1% – occur in only a small proportion of patients. Thus, the pathogenic relevance of altered function of the perturbed genes might not generalise beyond the small proportion of patients who carry them.

One approach to reconcile the issues of tractability and relevance is to systematically compare a variety of models that recapitulate different rare variants, thereby identifying phenotypic overlaps and convergent pathogenic mechanisms. Each variant represents an aetiologically distinct subpopulation of schizophrenia, thus capturing genetic heterogeneity. This approach has been applied to phenotypes of brain structure and functional connectivity in genetic mouse models of autism in order to investigate how disparate aetiologies all enhance the risk for autistic phenotypes (Ellegood et al., 2015; Zerbi et al., 2021). A complementary approach has recently been provided by two large-scale, well-powered and collaborative genomic studies (Singh et al., 2022; Trubetskoy et al., 2022) that identified several genes in which both common and rare variants show strong association with schizophrenia.

Trubetskoy et al. (2022) describes a GWAS of 76,755 schizophrenia cases and 243,649 unaffected control subjects that identified common variant associations at 287 distinct genomic loci, each having a small individual contribution to the risk of schizophrenia (median odds ratio of <1.05). Statistical fine-mapping prioritised 120 genes most likely to underlie associations at some of these loci (Trubetskoy et al., 2022). The companion exome sequencing study comprising 24,248 schizophrenia cases and 97,322 unaffected control subjects identified rare heterozygous coding variants associated with schizophrenia at the exome-wide significance level in ten genes (odds ratios between 3 and 52) and at a false discovery rate of <5% in a further 22 genes (odds ratios between 2 and 28) (Singh et al., 2022). Each gene represents a tractable entry point to elucidating biological mechanisms of this heritable disorder. Notable among the top hits in the study by Singh et al. (2022) are four genes (i.e. *FAM120A, GRIN2A*, *SP4*, *STAG1*) that were also among the 120 prioritised in the schizophrenia GWAS (Trubetskoy et al., 2022). Of particular interest is *GRIN2A*, which encodes the glutamate ionotropic receptor NMDA type subunit 2A (GRIN2A) of the NMDA receptor (NMDAR) (Box 1), a glutamate-activated ion channel that is eminently druggable, with positive and negative allosteric modulators available for preclinical studies (Hackos et al., 2016; Strong et al., 2014).

**Box 1. Schizophrenia risk gene *GRIN2A***

***GRIN2A* variants in other neurodevelopmental disorders**

**Fig. 1.**
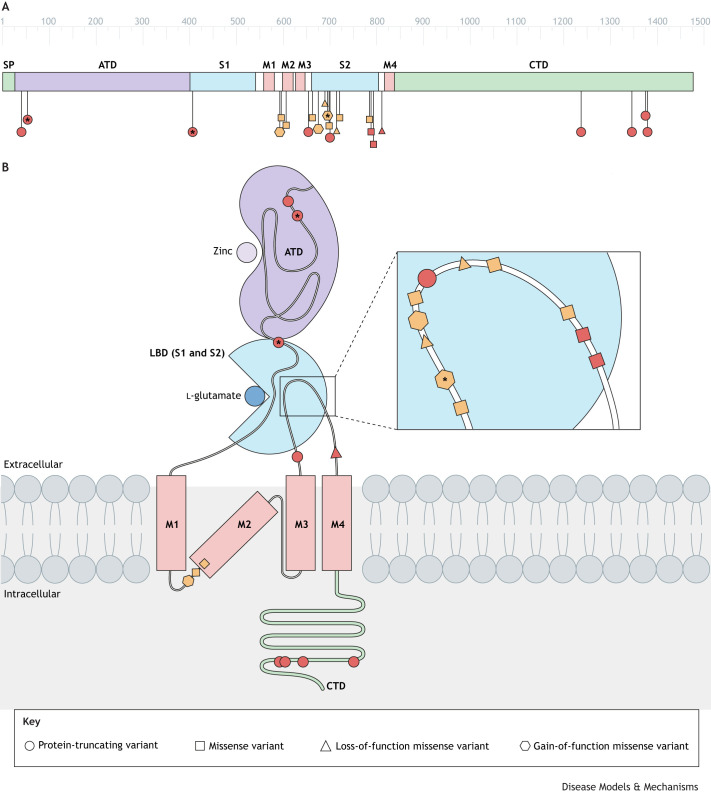
**Schizophrenia-associated variants in GRIN2A.** (A) Schematic linear representation of GRIN2A with 23 pathogenic variants that were exclusive to schizophrenia in the case-control analysis by Singh et al. (2022). High-risk variants (red) have an odds ratio of 24.1 and lower-risk variants (orange) have an odds ratio of 2.37. Protein-truncating variants (PTVs) are depicted as circles. *In vitro* functional testing of some missense variants revealed that three are likely to have loss-of-function effects (triangles), whereas another three are likely to have gain-of-function effects (hexagons) (CFERV, 2022; Swanger et al., 2016). Missense variants that had not been tested or for which no detectable effect was found upon testing are indicated by squares. One PTV and two missense variants (all indicated by *) had previously been identified in childhood-onset neurodevelopmental disorders (Fainberg et al., 2016; Kaplanis et al., 2020; Lesca et al., 2013; Strehlow et al., 2019). The scale (top) indicates the number of residues in the translated polypeptide (based on Uniprot Q12879). Linker regions are shown in white. (B) Shown in the approximate topological structure of GRIN2A is the location of variants that are likely to be pathogenic. Eleven (48%) of the 23 variants cluster in the S2 lobe of the ligand-binding domain that is involved in the binding of L-glutamate (magnified box). The ligand zinc is shown to bind the ATD. Panel B is adapted from Liu et al. (2021). ATD, amino-terminal domain; CTD, C-terminal domain; M1–M4, transmembrane domains 1–4; S1 and S2, lobes of the ligand-binding domain; SP, signal peptide.

Among the 23 rare, likely to be pathogenic variants in *GRIN2A* that were exclusive to schizophrenia cases in the case-control analysis by Singh et al. (2022), three had previously been identified in childhood-onset neurodevelopmental disorders (Fig. 1). This convergence implies some shared genetic risk and pathogenic mechanisms. It also aligns with the hypothesis that these conditions lie on a neurodevelopmental continuum that reflects a gradient of developmental disturbance, with greater prenatal impacts in early-onset neurodevelopmental disorders than in post-pubertal schizophrenia (Owen and O'Donovan, 2017).

**Functional consequences of rare *GRIN2A* variants**

*In vitro* assessment in heterologous expression systems of nine of the schizophrenia-associated rare *GRIN2A* variants revealed that they have diverse functional ramifications for GRIN2A-containing NMDARs (Fig. 1). Examination of the synaptic effects of epilepsy-associated missense variants in *GRIN2A* with contrasting loss-of-function (LoF) and gain-of-function (GoF) effects on NMDARs revealed that they lead to similar aberrant NMDAR-mediated synaptic currents when expressed in cultured CA1 pyramidal neurones, albeit via different mechanisms (Elmasri et al., 2022). This common synaptic effect hints at how both LoF and GoF *GRIN2A* variants might lead to manifestation of epileptic phenotypes. Further characterisation of the schizophrenia-associated *GRIN2A* variants is yet to be undertaken.

**Genome-based personalised medicine**

A 12-year-old girl with electroencephalogram (EEG) abnormalities and acoustic hallucinations, carrying the *GRIN2A* protein-truncating variant (PTV) A61Gfs*78, experienced improvement in these symptoms without side effects upon treatment with l-serine, the precursor of d-serine, a potent NMDAR co-agonist (Krey et al., 2022). A 9-year-old boy affected by infantile-onset epileptic encephalopathy with cognitive impairment, carrying the *GRIN2A* GoF missense variant L812M, experienced improvement in epileptic symptoms but unchanged cognitive ability upon treatment with memantine (Pierson et al., 2014), a well-tolerated anti-competitive NMDAR antagonist approved for the treatment of Alzheimer's disease. These cases suggest that l-serine or memantine may, potentially, be beneficial for schizophrenia patients with *GRIN2A* PTV or GoF missense variants, respectively. Careful profiling to confirm the type of molecular dysfunction will be required for stratification of patients and future effective personalised treatment strategies.

This robust genetic convergence of rare and common variant associations of these genes strongly supports their pathogenic role of perturbed function in schizophrenia, making them compelling candidates for further biological investigation in CRISPR/Cas9-mediated knock-in mice (Nagahama et al., 2020). It also illustrates how different types of genetic variation affecting the same gene can influence disease risk.

## Longitudinal studies across developmental stages

Schizophrenia is usually diagnosed in young adults at the time of the first episode of psychosis. However, converging evidence from epidemiological, brain imaging and neuropathological studies has led to widespread acceptance of the neurodevelopmental hypothesis of schizophrenia, first expounded in the 1980s (Murray and Lewis, 1987; Weinberger, 1987). This postulates that the illness is the end state of abnormal neurodevelopmental processes, caused by genetic and environmental factors, which begin years before the brain approaches its adult anatomical state in puberty. Although development is a continuous process, the brain is particularly vulnerable to insults (genetic and environmental) during the prenatal/perinatal period and, subsequently, during adolescence, a period of extensive remodelling of the brain circuitry (Jaaro-Peled and Sawa, 2020).

Juveniles at clinical high risk for psychosis (CHR-P) (Fusar-Poli, 2017) have been identified and included in large-scale longitudinal cohort studies. These studies have revealed that individuals who later develop schizophrenia exhibit subtle social and cognitive deficits (Mohn-Haugen et al., 2022; Tarbox and Pogue-Geile, 2008) – alongside reductions in prefrontal cortical activation (Smieskova et al., 2010) and frontal–temporal grey-matter volume (Fusar-Poli et al., 2011) – years prior to onset of psychosis. Current individualised prognostic models for detecting CHR-P individuals in the general population and predicting their transition to psychosis demonstrate, at best, only moderate prognostic accuracy (Fusar-Poli et al., 2015; Malda et al., 2019). However, this might be enhanced by incorporating the sum of risk-associated alleles at common variants across the genome, a so-called polygenic risk score that, currently, accounts for ∼8% of the variance in disease risk (Perkins et al., 2020; Trubetskoy et al., 2022). Improved understanding of the pathophysiological processes underlying the long-term progression to first-episode psychosis during the prodromal phase could advance our capacity to identify individuals at risk and impede disease progression by prophylactic intervention (Insel, 2010).

To further investigate the antecedents of schizophrenia, additional longitudinal studies of birth cohorts, genetic or familial high-risk populations and CHR-P populations are clearly important. However, they require large sample sizes, are affected by high drop-out rates and, inevitably, take a long time (Addington et al., 2015; Mollon et al., 2018). The prominent differences that exist between rodents and humans notwithstanding (Walker and Goldsmith, 2022), mice – with a generation time of only 9-11 weeks (Phifer-Rixey and Nachman, 2015) – offer a more expeditious platform for the longitudinal assessment of progressive brain changes and their relationship to the emergence of behavioural abnormalities.

Despite the growing focus on early detection, most published studies of mice harbouring schizophrenia-associated genetic variants have characterised behaviour only in young adults at 8-16 weeks of age (e.g. Nagahama et al., 2020), the murine equivalent of the typical age of onset. Whereas some psychotic symptoms, such as delusions and hallucinations, are not measurable in animals (Canetta and Kellendonk, 2018), a variety of tests with translational relevance to domains of psychopathology in schizophrenia have been employed. These include tests with exact parallels in humans, such as prepulse inhibition of startle reflex and the Iowa gambling task (Chadman et al., 2009; Forrest et al., 2014; Openshaw et al., 2022; Powell and Miyakawa, 2006). This Editorial does not critically evaluate these behavioural tests – Spark et al. (2022) does that superbly – but restricting analyses to adult mice foregoes important information on the behavioural phenotype during development.

To determine whether potential abnormalities follow a developmental trajectory, behaviour could be studied over a developmental time course, with repeated assessment of the same animals at appropriate ages (pup, juvenile, adult). To evaluate behaviour in adolescent mice, modified versions of adult behavioural tests have been developed (Eltokhi et al., 2020) but are not widely employed. In pups, a few assays are available to assess sensory-motor function, ultrasonic vocalisation and learning (Branchi and Ricceri, 2002; Michetti et al., 2022). By looking for correlations across ages, it would be possible to determine whether pup and juvenile behaviours predict adult behaviour. This is pertinent to understanding the developmental origin and early behavioural signs of schizophrenia but remains a neglected area of mouse model research.

Published analyses across different developmental stages include a longitudinal study of mice heterozygous null for neurexin-1α (Armstrong et al., 2020), the synaptic adhesion molecule that is a risk factor for schizophrenia when deletion variant 2p16.3 is present (odds ratio of 14.4) (Marshall et al., 2017). At 2 days of age, neurexin-1α heterozygous pups emitted fewer complex ultrasonic vocalisation calls than wild-type littermates upon separation from the dam and siblings. At 4 weeks of age, neurexin-1α heterozygous males, but not females, exhibited a significant reduction in duration of social sniffing during juvenile play testing. At 9 weeks of age, male neurexin-1α heterozygotes also showed less social sniffing along with increased aggression during adult social investigation testing, whereas olfactory habituation testing revealed no olfactory deficits (Armstrong et al., 2020).

Behaviour could also be assessed after weaning by automated home-cage monitoring that records the spontaneous behaviour of mice in social groups inside the home cage throughout the circadian cycle, with minimal human interference (Klein et al., 2022). By using this approach, 7–9-week-old mice hemizygous for a duplication corresponding to the human 16p11.2 duplication within chromosome 16 (16p11.2 *dp*/+ mice), which increases the risk for schizophrenia (odds ratios between 9.4 and 10.79) (Chang et al., 2017; Marshall et al., 2017), were housed in groups of three and monitored over a 3-day period. Relative to wild-type littermates, 16p11.2 *dp*/+ mice exhibited reduced locomotor activity, increased distance to their closest cage-mate during the dark phase, and reduced time spent in close proximity to cage-mates during the light phase (Bristow et al., 2020). Analysis of home-cage behaviour is being facilitated by the application of recent advances in machine learning and computer vision, to extract behavioural measurements from video footage (Mathis and Mathis, 2020). Technological advances are also permitting the combination of home-cage behaviours with electroencephalography, *in vivo* electrophysiology and Ca^2+^ imaging, to gain additional insights regarding the underlying circuits that drive behavioural outcomes (Mingrone et al., 2020).

*In vivo* neuroimaging of the same mice at different developmental time points can determine whether mouse models exhibit progressive structural brain abnormalities. *In vivo* structural magnetic resonance imaging of *Grin2a* heterozygous null mice – that model *GRIN2A* protein-truncating variants associated with schizophrenia (odds ratio of 18.1) (Singh et al., 2022) – has identified brain structures that have altered relative volumes at specific developmental stages (Salmi et al., 2018). *Grin2a* heterozygotes exhibited a decrease of the right cerebral cortex at 2 weeks of age, enlargement of both hippocampi and of the right corpus callosum at 4 weeks, and enlargement of the right hippocampus at 8 weeks (Salmi et al., 2018). Alas, Salmi et al. (2018) did not do behavioural testing on the same cohort of mice. However, conducting longitudinal neuroimaging and behavioural analyses in the same mice would allow a direct correlation between the imaging and behavioural findings, and would, in turn, allow to assess whether adult behavioural outcomes are predicted by early changes in the brain. This might identify potential targets for intervention to halt the aberrant neurodevelopment and the emergence of psychosis.

## Concluding remarks

Despite decades of research, schizophrenia remains a debilitating illness that is inadequately treated by current medications. To gain a better understanding of the biology and mechanisms of the disorder, the *in vivo* effects of rare large-effect variants identified by exome sequencing are widely studied in adult genetically-altered mice. However, this approach disregards the common small-effect variants that have been identified in GWAS and neglects the phenotype during development. Future studies need to compare the behaviour and neuroanatomy of a range of mouse models developed in response to the best available human genetic evidence, including genes implicated by both rare and common variants. Additionally, taking varying developmental time points into account will help to improve translation of outputs to clinical application. To potentiate the successful translation of such research, *Disease Models & Mechanisms* encourages collaboration and communication between fundamental and clinical researchers. We aim to support cutting-edge research that strives to address key challenges for the biological psychiatry field (Derks et al., 2022), with the aim of improving clinical outcomes for patients.
